# Endogenous Endophthalmitis successfully treated with Intravitreal Povidone-iodine injection: a case report

**DOI:** 10.1186/s12886-020-01487-w

**Published:** 2020-06-05

**Authors:** Hiroki Tanaka, Hiroyuki Nakashizuka, Yoshinobu Mizuno, Takayuki Hattori

**Affiliations:** 1 Nishikasai Inouye Eye Hospital, Tokyo, Japan; 2 Edogawa Hospital, Tokyo, Japan; 3 Division of Ophthalmology, Department of Visual Sciences, Nihon University Hospital, Nihon University School of Medicine, Tokyo, Japan; 4 Department of Ophthalmology, Teikyo University School of Medicine, Tokyo, Japan

**Keywords:** Endogenous endophthalmitis, Intravitreal injection, Povidone iodine

## Abstract

**Background:**

The usefulness of povidone-iodine as an alternative to antimicrobial agents, for endophthalmitis, has recently been documented. We report a case of endogenous endophthalmitis successfully treated with intravitreal injection of povidone-iodine.

**Case presentation:**

An 88-year-old woman underwent small bowel bypass surgery for postoperative ileus following rectal cancer resection. She developed a fever during total parenteral nutrition and was diagnosed with gram-positive cocci bacteremia of central venous catheter origin. The patient was referred to our department with chief complaints of ocular pain, hyperemia and decreased vision in the right eye, which had manifested during the febrile period. The initial examination revealed the visual acuity in her right eye to be finger counting and that in her left eye 0.2. The right eye showed a severe inflammatory reaction in the anterior chamber, fibrin deposition, and hypopyon. The fundus was difficult to visualize. Endogenous endophthalmitis due to bacteria was diagnosed.

Surgical treatment was judged to be difficult based on the patient’s poor general condition and mental status, and intravitreal injection of 0.1 ml of 1.25% povidone-iodine was performed on the same day. The inflammation rapidly diminished, and the hypopyon had disappeared 4 days after treatment. The fundus became visible 7 days after treatment and there was no recurrence of endophthalmitis findings. The visual acuity in her right eye recovered to that in the left eye (0.2).

**Conclusion:**

Intravitreal injection of povidone-iodine is potentially useful and effective as an alternative treatment of antibiotics for endogenous endophthalmitis patients, especially in whom surgical therapy is difficult.

## Background

Endogenous endophthalmitis is a severe eye infection that can rapidly lead to irreversible blindness. In cases with severe and sight-threatening endogenous endophthalmitis, vitrecomy is the preferred treatment strategy. However, vitrectomy is not applicable to all patients, because some are in poor systemic condition. Therefore, intravitreal antimicrobial therapy has greater clinical significance for endogenous endophthalmitis than for postoperative endophthalmitis. Furthermore, there is a broad range of causative organisms including both bacterial and fungal species. Recently, multidrug-resistant bacteria and vancomycin resistant bacteria have also been reported in endogenous endophthalmitis [1, 2]. In addition, vancomycin-associated hemorrhagic occulusive retinal vasculitis was reported following prophylactic use of an intracameral injection for cataract surgery [3]. Therefore, we need to develop antimicrobial agents that have broad-spectrum anti-bactericidal and anti-fungal actions even against multidrug-resistant bacteria.

The usefulness of povidone-iodine (PI) as an alternative to antibiotics for the treatment of endophthalmitis has been experimentally investigated, and the intravitreal concentrations of PI, safe for ocular tissues and effective for treating endophthalmitis, have been calculated to range from 0.013 to 0.027% [4, 5]. Furthermore, the usefulness of vitrectomy for endophthalmitis employing 0.025% PI irrigation [6] and that of 1.25% PI/0.1 ml intravitreal injection (IVI) as the initial treatment for endophthalmitis have also been reported [7].

We herein present a case of endogenous endophthalmitis successfully treated with IVI of PI.

## Case presentation

An 88-year-old female underwent small bowel bypass surgery for postoperative ileus following rectal cancer resection and was receiving care in the surgical department of Edogawa Hospital. Postoperatively, the patient developed a fever of unknown origin and was diagnosed with bacteremia associated with a central venous catheter. After catheter removal, cefmetazole sodium 1 g/day was started.

**Fig. 1:**
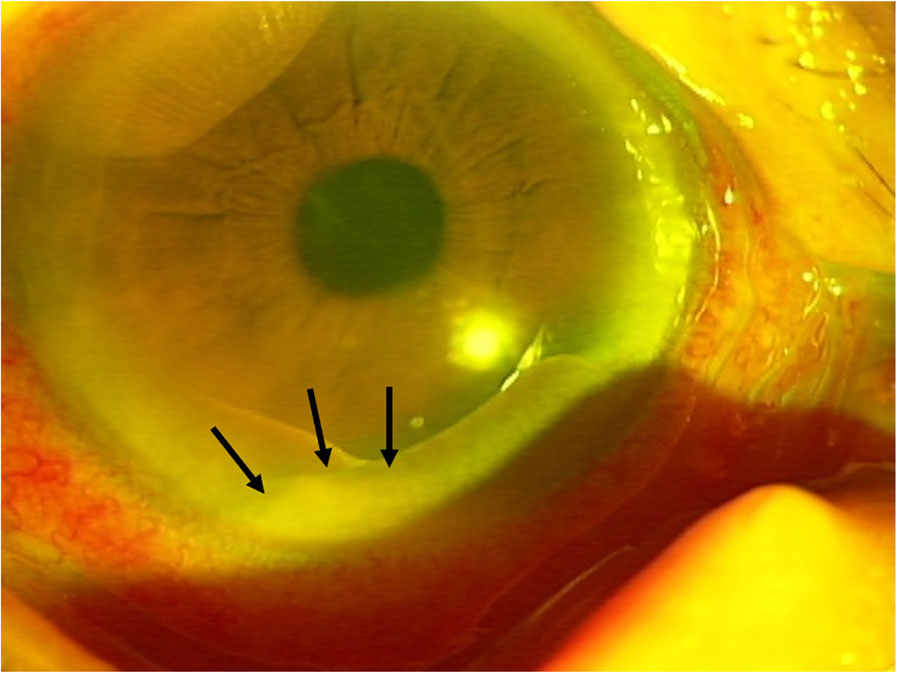
Anterior segment photograph obtained at the first visit. Conjunctival hyperemia and marked inflammation and hypopyon in the anterior chamber (arrow) were noted on the first examination. This patient also has arcus senilis on the peripheral cornea

**Fig. 2:**
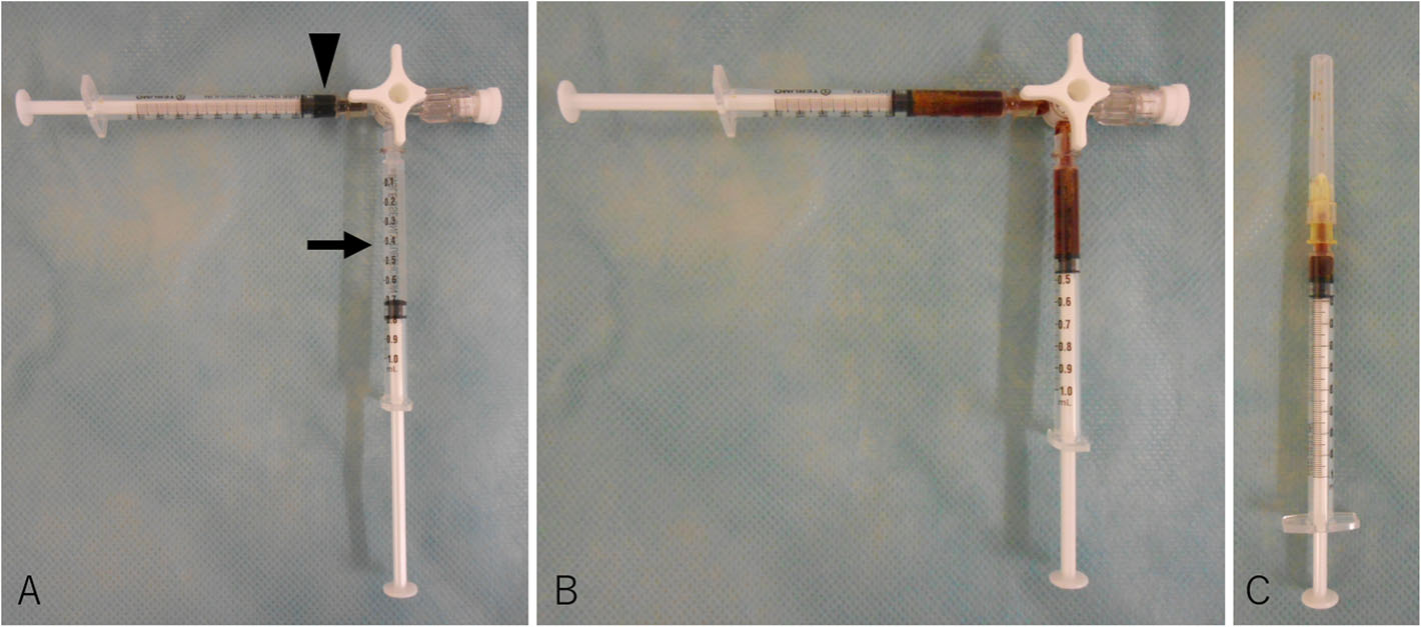
Adjustment of 1.25%/0.1 ml for IVI of PI. A. 0.1 ml of 10% PI (arrowhead), an undiluted solution, and 0.7 ml of saline solution (arrow) are prepared. B. These are mixed and diluted to achieve a concentration of 1.25%. C. 1.25% / 0.1 ml of PI is then injected into the vitreous

She had conjunctival hyperemia and decreased visual acuity in the right eye during the febrile period, and she was thus referred to the ophthalmology department on August 10th, 2017. The initial examination showed visual acuity in the right eye to be finger counting, that in the left eye 0.2. Slit-lamp examination revealed conjunctival hyperemia in the right eye, as well as a marked inflammatory reaction in the anterior chamber with hypopyon (Fig. 1). Moderate cataract was also noted, but the ocular fundus could not be visualized in detail due to poor visibility caused by vitreous opacity. As gram positive cocci were detected by blood culture, the endogenous endophthalmitis was attributed to these bacteria. However, not only surgical treatment but also repeated intravitreal injections and even a vitreous tap were judged to be difficult based on her poor general condition and mental status, including dementia. Furthermore, the details of the gram-positive cocci detected from blood culture and the antibiotic sensitivities were still being examined at the time. Thus, after written informed consent had been obtained from her daughter, we selected IVI of 1.25% / 0.1 ml PI, which has broad-spectrum efficacy including against antibiotic-resistant bacteria, instead of IVI of antibiotics. This treatment was performed by one of the authors (H.N.). Adjustment of 1.25% / 0.1 ml for IVI of PI was conducted as follows; 0.1 ml of 10% PI, which is an undiluted solution, and 0.7 ml of saline solution are mixed and diluted, and 0.1 ml is then injected into the vitreous (Fig. 2). This treatment was approved by the Ethics Committee of Nihon University Hospital. Levofloxacin 6 times daily, tropicamide 3 times daily and atropine 1 time daily were also instilled, and the antibacterial venous infusion was changed to cefepime hydrochloride 1 g daily after the gram-positive cocci were revealed to be *Staphylococcus aureus* and the antibiotic sensitivities were determined.

**Fig. 3:**
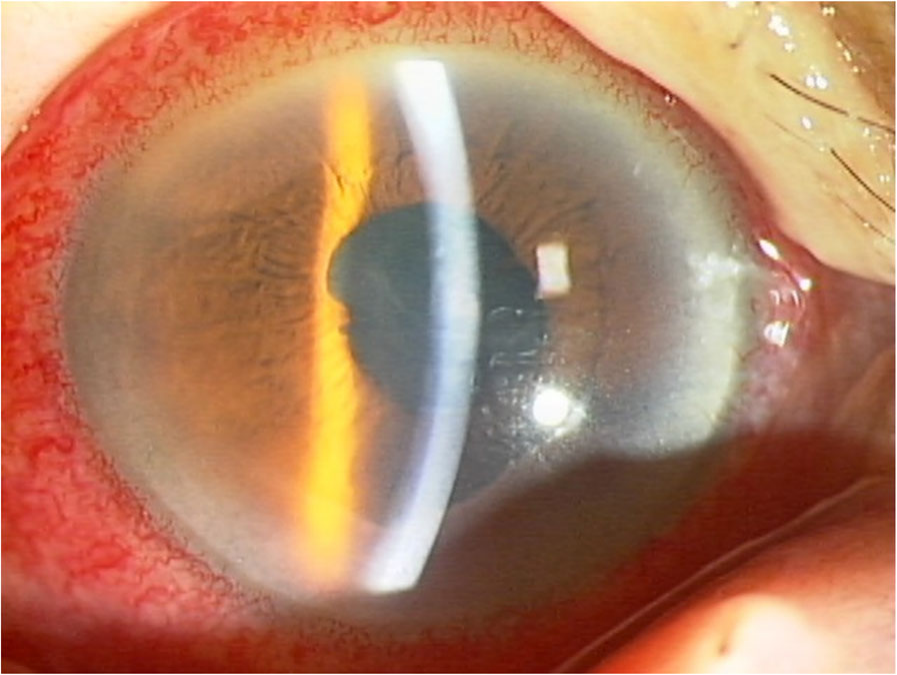
Anterior segment photograph obtained 7 days after IVI. The inflammation in the anterior chamber rapidly diminished, resulting in improved transparency of the ocular fundus

Two days after the IVI, the hypopyon was diminished and her ocular pain resolved. Then, 4 days after the IVI, the inflammation in the anterior chamber showed rapid resolution and the hypopyon had disappeared completely. The fundus was visible 7 days after the IVI (Fig. 3). The patient showed gradual recovery of her general condition, and there was no recurrence of endophthalmitis findings, such that she was discharged from our hospital 11 days after the IVI.

**Fig. 4:**
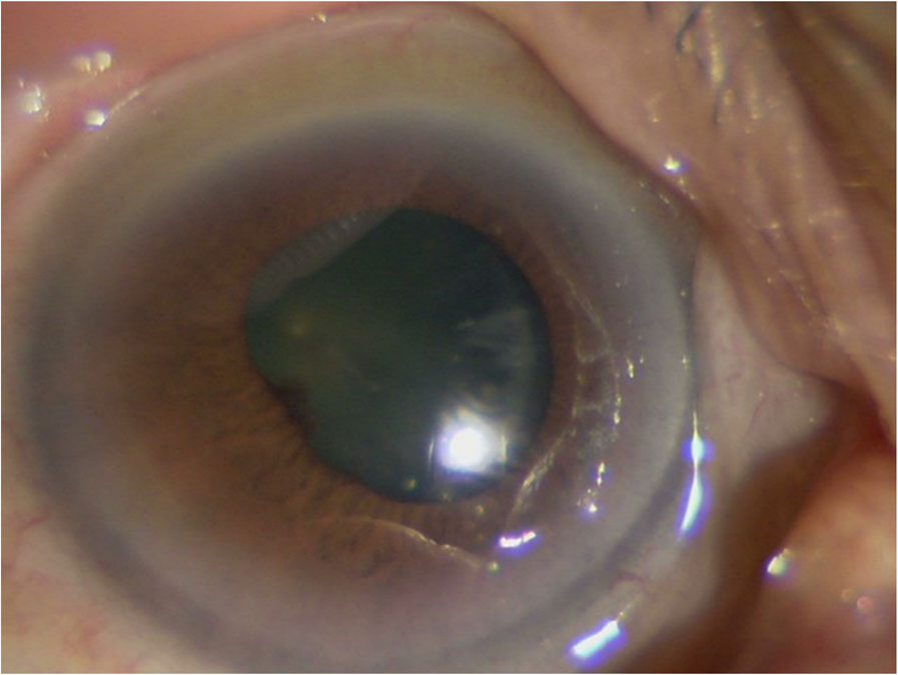
Anterior segment photograph obtained 1 month after IVI. The inflammation in the anterior chamber has completely disappeared and the right eye visual acuity has improved to 0.2, the same as that of the left eye.

One month after the IVI, the inflammatory findings had completely disappeared, and the visual acuity of the right eye (0.2) was the same as that of the left eye (Fig. 4). This condition was maintained for 4 months after treatment. She discontinued visiting our hospital thereafter due to deterioration of her systemic condition.

## Discussion and conclusions

The effects of IVI of PI on experimental endophthalmitis were reported by Kim et al. [8]. Four groups of rabbits treated with 0.1% / 0.1 ml and 0.3% / 0.1 ml PI once or three times every second day were compared for endophthalmitis caused by *Staphylococcus epidermidis*. Fourteen days later, 40% of the single dose group receiving 0.1% showed positive cultures and 30% of the single dose group receiving 0.3% showed positive cultures, in other words 60 and 70% of cases treated with IVI of 0.1 and 0.3% PI, respectively, were culture negative even with injection of a single dose, whereas all cases treated three times every second day showed culture negativity in both the 0.1% and the 0.3% group. Assuming the vitreous volume of the rabbit eye to be 1.5 ml, the intravitreal PI concentrations were estimated to be 0.0067 and 0.02% at 0.1% / 0.1 ml and 0.3% / 0.1 ml, respectively. The PI half-life was 3.3–3.6 h, and no retinal damage was detected by electroretinograms (ERGs) in any of the groups indicating PI three times every second day to be effective and safe for treating endophthalmitis.

In our present patient, a single IVI at a dose of 1.25%/0.1 ml PI was curative. Assuming the vitreous volume to be 5 ml, the intravitreal concentration of PI would be 0.025%, and this concentration has been confirmed to be safe for ocular tissues in previous experimental studies, with no abnormalities having been observed on ERGs or by histopathological examination [5]. Compared to the vitreous PI concentrations of 0.0067 and 0.02% reported by Kim et al. [8], the concentration of 0.025% used in our study was higher, and it is likely that high efficacy was thus obtained by a single administration. If endophthalmitis is unresponsive to the initial treatment, repeated administrations of 1.25% / 0.1 ml PI, possibly as many as three times every second day, might achieve efficacy.

Adjusting 1.25%/0.1 ml for IVI of PI is simple and rather easily achieved as compared with IVI of an antimicrobial agent. Furthermore, PI use has advantages including cost-effectiveness, that it can easily be obtained almost anywhere, and has broad-spectrum efficacy against resistant bacteria. Furthermore, it is said that vancomycin exerts a bacteriostatic effect after injection, though 8 h are needed for the bactericidal action to fully manifest [9], while PI requires only 15 s at low concentrations to kill infection-causing bacteria [10].

In our previous clinical study, 1.25% / 0.1 ml for IVI of PI was used as the primary treatment for endophthalmitis and was followed by vitrectomy using 0.025% PI Balanced Salt Solution Plus. No adverse events were detected on ERGs, by Goldmann perimetry, or on examination of corneal endothelial cells [7].

There are endogenous endophthalmitis cases not eligible for operative medical treatment due to poor general condition. In these cases, IVI of antibiotics should be selected as the main treatment as well as systemic antibiotic therapy. However, sampling the vitreous might be difficult and, even if a sample could be obtained, several days would be needed to obtain the details of the bacteria detected and to confirm antibiotic sensitivities. Furthermore, endogenous endophthalmitis caused by vancomycin resistant bacteria has also been reported [1, 2]. Thus, we consider IVI of 1.25% / 0.1 ml PI to be a potential treatment option, to be administered in selected cases, instead of IVI of antibiotics.

Our present patient’s general condition was quite poor, such that we could not perform detailed examinations. Thus, only visual acuity, slit-lamp microscopy and fundus examination were performed. Another shortcoming of this case report is that the quality of the anterior segment photographs is poor due to the patient’s facial instability. Based on the aforementioned experimental reports and our previous clinical study results [7], the vitreous concentration of PI used in this case appears to be safe for ocular tissues including the retina. However, the possibility of endophthalmitis causing corneal and retinal weakening and unexpected complications cannot be ignored. Meticulous follow-up is thus essential. Before recommending this treatment strategy for endophthalmitis patients in general, we need to accumulate more cases, with detailed examination findings such as those of corneal endothelial cells, ERG and perimetry.

This is the first report, to our knowledge, describing IVI of PI, administered without other treatments, as achieving full resolution of endophthalmitis.

## Data Availability

The datasets used and/or analyzed during the current study are available from the corresponding author upon reasonable request.

## Abbreviations

PI: povidone-iodine
IVI: intravitreal injection
ERG: electroretinogram
